# The London School of Tropical Medicine

**Published:** 1901-10-26

**Authors:** 


### The Institutional Workshop.

### THE LONDON SCHOOL OF TROPICAL MEDICINE.

Lord Brassey delivered the inaugural address at the opening of the third winter session of the London School of Tropical Medicine at the Royal United Service Institution, Whitehall, on October 16th. Mr. Percival Nairne presided.

Lord Brassey said the meeting had been called for the purpose of obtaining funds for carrying on the work of the school. The salient facts were these: The school was established on the initiative of Mr. Joseph Chamberlain, through whose influence the sum of £16,000 was raised. The fact that of this sum £3,500 was contributed by the Colonial Office, and £200 by the Foreign Office, showed, he thought, that the necessity of such a school was realised by those most competent to give an opinion. These facts should be commended to the public. The school was originally intended for the instruction of surgeons in the Colonial and Indian service, but it had not been confined to these. At first provision was made for about twelve students, and it was believed that this would be sufficient. It had been shown, however, that the demand was much greater than had been anticipated; in the second session the average attendance at lectures and classes was 25, and students had had to be refused admission. Four or five had postponed their special training until vacancies occurred. Funds were wanted also for the endowment of chairs for teachers; and it was desired to place the school on a sound financial basis altogether. The importance of the health of Europeans holding office in tropical countries could not be over-estimated, since so much depended upon their efficiency. It was obvious that it would greatly conduce to this efficiency if the absences from work could be diminished; it was an all-important thing that men should not be absent from their posts. There was another point to which he must refer, viz., the Seamen's Hospital Society had accepted the offer of Sir Francis Lovell's services: he was about to proceed to the tropics to raise funds for the school, and great hopes were entertained of the beneficial results of his mission. It was his (the speaker's) duty to ask the public for the large contribution of £100,000, which was necessary if the whole design was to be carried out successfully. Large as it was, it was yet small when they realised how great was the Empire within the tropics, and that the object of the school was to improve the hygienic and sanitary conditions of fellow-subjects. In conclusion he would say that this was an institution that could not stand still; the choice was between going backward and going forward; and he thought that when the considerations upon which he had touched were weighed, the public could not fail to give a favourable response to his appeal. He advocated a forward and progressive policy in the interests of Europeans in the tropics.

Dr. Patrick Manson, who followed, said the school had two objects: (1) The education of the practitioner, and (2) the advancement of mental science as regarded tropical diseases. How far these objects could be carried out depended on the public. The school up to the present had proved a distinct success; session after session the applications for admission had increased, and this was a little confusing and troublesome. It was a pity that an institution should be checked by a superfluity of work, as must be the case until the school was able to cope successfully with the demands made upon it. It was a pity also that any student wishing to devote himself to the study of a particular branch of medical science should have to give up the idea because there was not room for him. Those who came were no callow youths in want of something to do: some were grey-haired men of long experience. A couple of dozen of these full-grown men had to work in a laboratory that was too small for them; lectures were given in the dining-room, a quite unsuitable place; specimens that should be collected in a museum were scattered over the establishment; there was as yet no good library, and the living accommodation was inadequate. The six bedrooms were always occupied. The advantage of living on the premises was great: it saved time, and insured to the students opportunities of seeing acute changes in the condition of patients, which frequently took place in the night. Many of of the students came from foreign countries; they knew no one in London, and the school offered them a home. Round the mess table ideas were exchanged, and a man left the school carrying with him not only his own share of knowledge, but the benefit of two dozen other men's knowledge and experience as well. It had been forced upon the committee that a centre was wanted through which special inquiries might be answered. Mercantile companies were applying to the school for information on tropical diseases, and they were also asked to supply medical men. This had not been foreseen, and he thought that in the future it would form an important part of the work. At the outset they had been told that the special education the school proposed to give was already supplied at the ordinary medical schools. People who said this were unacquainted with the ordinary medical schools. He had already adduced, in an address at St. George's Hospital, a number of instances, which led to fatal results, proving the need for special knowledge such as was not given at the ordinary schools; these instances had been called "figments of the imagination." His statement, for example, that beri-beri was not uncommon in the London Docks was contradicted. But only the other day a negro was brought into the Seamen's Hospital suffering from what anyone acquainted with tropical diseases would at once have known to be beri-beri. It was not recognised as such by the house surgeon, who believed there was some foreign matter in the man's throat, and that he was choking. He took him upstairs, but the patient died before he could be got to bed. A knowledge of tropical diseases would have saved the man's life, and such cases occurred much more frequently than people imagined. Another thing which the committee keenly felt was the difficulty of promoting a knowledge of tropical medicine. The London School had contributed its quota to the science of medicine. It had been demonstrated that disease was conveyed by mosquitoes. Dr. George Lowe had contributed some very important facts; he had shown that 11 or 12 per cent. of the inhabitants of the Barbadoes islands were suffering from disease. He had also shown how it was possible to remove elephantiasis and cognate diseases in the course of one generation. That alone justified the existence of the school. The Government, he was glad to say, had encouraged Dr. Lowe's investigations. An expedition to the Brazils was organised last year, when two doctors went out to study yellow fever. One had fallen a victim to the disease; but the other, Dr. Durham, was so firmly convinced of the necessity for this study that he was again going forth to brave death, and this time he would be accompanied by the speaker's son. If men could be found to expose their lives to such risks, the money should easily be forthcoming. Germany, as Professor Koch had told him, in reply to his inquiries, was spending from £1 to £2 a day on expeditions of medical research. He himself had been astonished at the liberality of Germany in this respect, and still more so at the lack of any corresponding enthusiasm for medical science in England. Germany spent five times as much as England on this object. Dr. Koch's letter, which was read by Dr. Manson, gave the names of the doctors sent by Germany to other countries to study disease: those in Europe received 20 marks a day and expenses; those outside, 40 marks, 1,000 marks being granted also in these cases for equipment. If England were to subsidise for the study of tropical diseases, the speaker continued, a hundred men might be at work. At the school the pittance for which the doctors worked was so poor that he would be ashamed to mention it.

Sir Francis Lovell then explained the circumstances under which he had undertaken the mission to which reference had been made. It was some thirty years since he first went abroad; on that occasion he had Lord Brassey as a fellow passenger, and he had met him since on board the *Sunbeam*. He was happy in being able to start on his mission under Lord Brassey's auspices. He had returned last April from abroad, and on doing so had told the Colonial Secretary that he contemplated retirement. It was then suggested that some arrangement with the Seamen's Hospital Society might be come to, to put the London School of Tropical Medicine on a firmer basis. Admirable work was being done there under Dr. Manson, but it was absolutely necessary that more funds should be forthcoming. The public at home had contributed very liberally, and it was felt that an appeal ought to be made abroad. He offered to visit some of the tropical stations, and to endeavour to gain the support of merchants and others. The Seamen's Society, after due consideration, accepted the offer, and he would shortly proceed to India, Ceylon, China, Japan, Australasia, etc., and it was his intention to do his utmost to get the London School put on a secure foundation as a teaching body in the great City of London.

Sir Charles Gage Brown having proposed a vote of thanks to Lord Brassey, who was obliged to leave almost immediately after his address, the meeting terminated.

